# Evaluation of Adhesion Properties of Thin Film Structure through Surface Acoustic Wave Dispersion Simulation

**DOI:** 10.3390/ma15165637

**Published:** 2022-08-16

**Authors:** Yu Min Choi, Dongchan Kang, Jeong Nyeon Kim, Ik Keun Park

**Affiliations:** 1 Department of Mechanical Engineering, Graduate School, Seoul National University of Science and Technology, Seoul 01811, Korea; 2 Graduate School of Nano IT Design Fusion, Seoul National University of Science and Technology, Seoul 01811, Korea; 3 Ginzton Laboratory, Stanford University, Stanford, CA 94305, USA; 4 Department of Mechanical and Automotive Engineering, Seoul National University of Science and Technology, Seoul 01811, Korea

**Keywords:** V(z) curve technique, dispersion characteristics, adhesion strength, scanning acoustic microscope

## Abstract

A theoretical simulation study of the dispersion characteristic of the surface acoustic wave (Rayleigh wave) was conducted by modeling the adhesion interlayer with stiffness coefficients to evaluate the bonding properties of nano-scale thin film structures. For experimental validation, a set of thin film specimens were fabricated—637 nm, 628 nm, 637 nm, 600 nm, and 600 nm thick titanium (Ti) films were deposited on silicon (Si) (100) substrate using a DC Magnetron sputtering process with DC power from 28.8 W, 57.6 W, 86.4 W, 115.2 W, and 144 W. The thicknesses of the Ti films were measured using a scanning electron microscope (SEM). Surface acoustic wave velocity for each of the manufactured thin film specimens was measured by using a V(z) curve technique of a Scanning Acoustic Microscope. The measured velocity, transducer frequency, and thickness of the film were applied to dispersion characteristic simulation for a given stiffness coefficient to calculate adhesion strength of each specimen. To verify the simulation result, the adhesion force of each specimen was measured using a nano-scratch test and then compared with the calculated values from the dispersion characteristic simulation. The value of adhesion strength from the dispersion characteristic simulation and the value of adhesion force of the nano-scratch test were found to have a similar tendency according to the process variable of the thin film. The results demonstrated that the adhesion strength of a thin film could be evaluated quantitatively by calculating the dispersion characteristics with the adhesion interlayer stiffness model.

## 1. Introduction

Thin film structures are used in various fields such as semiconductors, displays, and MEMS devices. As the technology advances, the thickness of the thin film becomes smaller on a micro/nano-scale. Research has been conducted to detect various defects and evaluate physical properties. In particular, since the adhesion strength of the thin film directly affects the life and performance of the product, various studies have been conducted to evaluate adhesion strength [1,2,3,4,5]. However, destructive techniques, such as SEM, TEM, nano-indentation test, and nano-scratch test, damage the specimen during pretreatment or measurement, so the specimen cannot be reused [5,6,7]. On the other hand, nondestructive techniques that use acoustic and optics to evaluate the adhesion strength have a limitation of non-derived quantitative results [2]. A study to increase reliability was conducted by using the destructive technique nano-scratch test and the non-destructive technique AE at the same time [3]. To overcome these limitations, this study was conducted to quantitatively evaluate the adhesion strength of thin films using an acoustic nondestructive technique. The advantage of non-destructive evaluation using acoustic is that it is possible to evaluate properties by measuring the acoustic velocity with high precision in a small area based on the acoustic–elastic effect, which is the relationship between the acoustic velocity propagating inside the material and the internal/applied stress. Based on this, properties of single and composite materials such as acoustic anisotropy, twin strain, processing change layer, thin film thickness measurement, residual stress, bonding properties, and mechanical properties can be identified [8,9,10,11,12]. For a nondestructive evaluation, the acoustic velocity of the Rayleigh wave was measured by applying the V(z) curve technique of a scanning acoustic microscope. Then, the dispersion characteristics of the surface acoustic wave (*SAW*) in a multilayer structure were simulated to convert the measured sound velocity to adhesion strength. The method of simulating the theoretical dispersion curve was carried out following that of the previous study [13]. In order to evaluate the adhesion properties of thin film, a virtual adhesion interface, having a spring stiffness coefficient between the thin film and the substrate, was modeled. Conducting the dispersion characteristic simulation that includes this modeling, the dispersion characteristics were calculated for the given stiffness coefficient. A titanium thin film was deposited to a silicon substrate using a DC magnetron sputtering process to verify the adhesion strength evaluation method through dispersion characteristic simulation. *SAW* velocity of the manufactured specimens were then nondestructively measured using a V(z) curve technique of the scanning acoustic microscope. The adhesion strength according to physical properties, such as measured sound velocity, the frequency used in the measurement, and the thickness of the thin film, was derived from the dispersion characteristic simulation. In order to verify the reliability of the adhesive strength evaluation method through dispersion characteristic simulation, a nano-scratch test, a commercial adhesive force evaluation technique, was performed on the fabricated specimens and the results were compared.

## 2. Related Theory

### 2.1. Dispersion Characteristics of Multilayer Thin Film

The ultrasound propagating through a multilayered thin film structure has a dispersion characteristic, in which the phase velocity changes according to the thickness and frequency of the thin film. To evaluate the dispersion characteristic, a dispersion curve for the thin film and the substrate was derived using Thomson–Haskell’s transfer matrix method [14]. The properties of the elastic wave traveling through the multilayered structure are expressed with the displacement–stress equation expressed in terms of amplitude. Applying this to the transfer matrix, the dispersion characteristics for the multilayered structure was calculated. The reliability of the dispersion curve calculated by the transfer matrix method was evaluated through previous studies [14,15,16].

**Figure 1:**
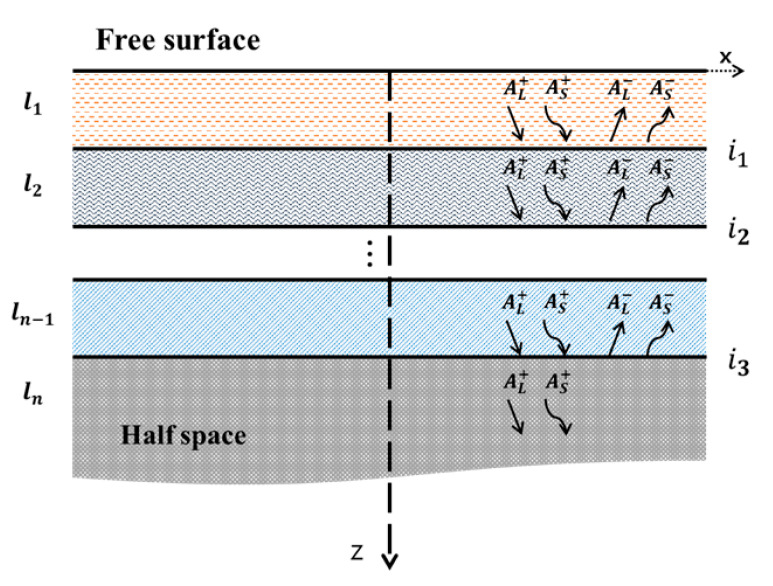
Schematic diagram of *SAW* propagating in multi-layer thin films.

Figure 1 shows the amplitude parameter through the multi-layered thin film structure. Wave analysis for multi-layer structures is generally performed in a two-dimensional coordinate system, because the wavelength is negligibly small compared to the width of the structure. In the *SAW* traveling through the thin film, only the propagation direction (*x*) and the depth (*z*) of the wave are considered and analyzed. In Figure 1, $A_{L}^{+}$ and $A_{S}^{+}$ are the upward longitudinal and transverse wave components, respectively; $A_{L}^{-}$ and $A_{S}^{-}$ are the downward longitudinal and transverse wave components. Therefore, the displacement vectors of the longitudinal and transverse waves, represented by the scalar function *ϕ* and the vector function *ψ*, are as shown in Equation (1) below.

(1) $$\begin{array}{c} {\left\{\begin{array}{c} u_{x}\\ u_{y}\\ u_{z} \end{array}\right\}}_{\left(L\right)}=\left\{\begin{array}{c} \frac{\partial }{\partial x}\\ \frac{\partial }{\partial y}\\ \frac{\partial }{\partial z} \end{array}\right\}\times \phi =\left\{\begin{array}{c} k_{x}\\ 0\\ k_{z} \end{array}\right\}A_{L}\;e^{i\left(kx-wt\right)}\\ {\left\{\begin{array}{c} u_{x}\\ u_{y}\\ u_{z} \end{array}\right\}}_{\left(S\right)}=\left\{\begin{array}{c} \frac{\partial }{\partial x}\\ \frac{\partial }{\partial y}\\ \frac{\partial }{\partial z} \end{array}\right\}\times \psi =\left\{\begin{array}{c} k_{x}\\ 0\\ -k_{z} \end{array}\right\}A_{S}\;e^{i\left(kx-wt\right)}\; \end{array}$$

Here, $k_{x}$ and $k_{z}$ are the wavenumber in the *x*-direction and *z*-direction, respectively, $A_{L}$ is the amplitude of the longitudinal wave, and $A_{S}$ is the amplitude of the transverse wave. In the Cartesian coordinate system, the relationship between the displacement, stress, and amplitude in the propagation direction (*x*-axis) and the depth direction (*z*-axis) is as shown in Equation (2) below.

(2) $$\left\{\begin{array}{c} u_{x}\\ u_{z}\\ \sigma_{zz}\\ \sigma_{xz} \end{array}\right\}=\left[\begin{array}{cccc} k_{x}g_{\alpha } & \frac{k_{x}}{g_{\alpha }} & C_{\beta }g_{\beta } & -\frac{C_{\beta }}{g_{\beta }}\\ C_{\alpha }g_{a} & -\frac{C_{\alpha }}{g_{\alpha }} & k_{x}g_{\beta } & \frac{k_{x}}{g_{\beta }}\\ i\rho Bg_{\alpha } & \frac{i\rho B}{g_{\alpha }} & 2i\rho k_{x}\beta^{2}C_{\beta }g_{\beta } & \frac{2i\rho k_{x}\beta^{2}C_{\beta }}{g_{\beta }}\\ 2i\rho k_{x}\beta^{2}C_{\alpha }g_{\alpha } & \frac{-2i\rho k_{x}\beta^{2}C_{\alpha }}{g_{\alpha }} & i\rho Bg_{\beta } & \frac{i\rho B}{g_{\beta }} \end{array}\right]\cdot \left\{\begin{array}{c} A_{L}^{+}\\ A_{L}^{-}\\ A_{S}^{+}\\ A_{S}^{-} \end{array}\right\}$$

Here, the amplitude of the ultrasonic wave traveling in the depth direction of the thin film structure is represented by + subscript, and that traveling in the opposite direction is represented by the—subscript. The variables, $C_{\alpha },\;C_{\beta },\;g_{\alpha },\;g_{\beta }$ and B, are arbitrarily designated abbreviations for convenience of calculation and are expressed as follows.

(3) $$\begin{array}{c} C_{\alpha }={\left(w^{2}/\alpha^{2}-k_{x}^{2}\right)}^{1/2}\\ C_{\beta }={\left(w^{2}/\beta^{2}-k_{x}^{2}\right)}^{1/2}\\ g_{\alpha }=e^{i{\left(w^{2}/\alpha^{2}-k_{x}^{2}\right)}^{1/2}z}\\ g_{\beta }=e^{i{\left(w^{2}/\beta^{2}-k_{x}^{2}\right)}^{1/2}z}\\ B=w^{2}-2\beta^{2}k_{x}^{2} \end{array}$$

The 4 × 4 matrix in Equation (2) is defined as a coefficient matrix [*D*], which represents the relationship between displacement–stress and amplitude. In a multilayer thin film with n layers, the relationship between amplitude and displacement/stress is defined by the coefficient matrix [*D*], which can be expressed by Equation (4).

(4) $${\left\{\begin{array}{c} A_{L}^{+}\\ A_{L}^{-}\\ A_{S}^{+}\\ A_{S}^{-} \end{array}\right\}}_{ln}={\left[D\right]}_{ln,t}^{-1}{\left[D\right]}_{ln-1,b}{\left[D\right]}_{ln-1,t}^{-1}\cdots {\left[D\right]}_{l1,b}{\left[D\right]}_{l1,t}^{-1}\cdot {\left\{\begin{array}{c} u_{x}\\ u_{z}\\ \sigma_{zz}\\ \sigma_{xz} \end{array}\right\}}_{l1.t}$$

Here, the coefficient matrix of bottom and top interfaces are represented by *b* and *t* subscripts. Applying the transfer matrix method, the *SAW* velocity according to the thickness and frequency of the thin film can be derived.

### 2.2. Modeling the Adhesion Interlayer

The dispersion characteristics algorithm in Section 2.1 managed to derive the theoretical dispersion curve in the perfect bonding state between the thin film and the substrate. However, for the actual thin film deposited, variables such as properties of the substrate and the thin film, process equipment used, pretreatment conditions, and the deposition parameters determine the adhesion properties of the interface. Therefore, there will be some discrepancies between the theoretically calculated results and the actual results. In order to accurately evaluate the properties of the micro/nano-scale thin films, it is necessary to derive the dispersion characteristics considering the adhesion properties between the thin film and the substrate.

In order to consider the imperfect bonding state existing in the actual specimen, the adhesion properties were evaluated by modeling a virtual interface that has a spring stiffness coefficient value [4,17,18,19]. If the displacement of the interface in the perfect bonding state is $\Delta u_{p}$, and the displacement in the imperfect bonding state where defects or porosity exist at the interface is $\Delta u_{I}$, the total displacement, $\Delta u_{T}$, can be expressed as follows, when tensile or compressive stress is applied. Equation (5) is derived displacement focusing on the state of adhesive properties, and other parameters are not considered.

(5) $$\Delta u_{T}=\Delta u_{P}+\Delta u_{I}$$

At the imperfect bonding state, the stiffness coefficient of the interface between the thin film and the substrate under a stress condition can be expressed as in Equation (6) below.

(6) $$k=\sigma /\Delta u_{I}$$

It was theoretically confirmed that the more imperfect the bonding state was, the larger the displacement and the smaller the spring stiffness coefficient. In the thin film structure, the correlation between the stiffness coefficient and the adhesion force was identified through quasi-static modeling based on the fracture mechanics [17]. In order to apply the stiffness coefficient of the interface defined in Equation (6) to the dispersion characteristic simulation, the velocity of the longitudinal and transverse waves, respectively, can be expressed as follows.

(7) $$C_{L}=\sqrt{\frac{K_{N}d_{i}}{\rho_{i}}}\;,\;\;\;\;C_{S}=\sqrt{\frac{K_{T}d_{i}}{\rho_{i}}}$$

In Equation (7), $K_{N}$ and $K_{T}$ represent the stiffness coefficients in the normal and tangential directions, and $d_{i}$ and $\rho_{i}$ represent the thickness and density of the interfacial layer. The thickness of the bonding interface layer was set to 4% of the thickness of the thin film and the density was set to 11% of the lowest density among the thin film and the substrate [4]. Through this, the transverse and longitudinal wave velocities of the ultrasonic wave traveling through the interfacial layer can be calculated with the spring stiffness coefficient. The dispersion characteristic simulation considering the bonding layer model can be derived as Equation (8) by modifying Equation (4).

(8) $${\left\{\begin{array}{c} A_{L}^{+}\\ A_{L}^{-}\\ A_{S}^{+}\\ A_{S}^{-} \end{array}\right\}}_{ln}={\left[D\right]}_{sub,t}^{-1}{\left[D\right]}_{lnter,b}{\left[D\right]}_{lnter,t}^{-1}{\left[D\right]}_{film,b}{\left[D\right]}_{film,t}^{-1}\cdot {\left\{\begin{array}{c} u_{x}\\ u_{z}\\ \sigma_{zz}\\ \sigma_{xz} \end{array}\right\}}_{l1.t}$$

**Figure 2:**
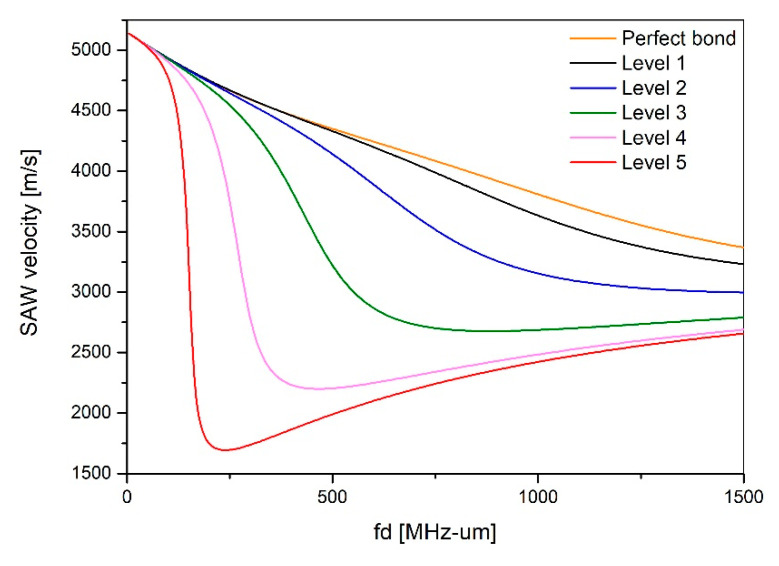
Dispersion curve simulation according to interface conditions.

Here, the sub, inter, and film denoted by subscripts represent substrates, inter-layer, and thin films. In order to derive the dispersion characteristics according to the stiffness coefficient of the bonding interface, five levels of adhesion strength were established, as shown in Table 1. The dispersion curve derived by performing the dispersion characteristic simulation according to the adhesion level set in Table 1 is shown in Figure 2.

The results show that the higher the stiffness coefficient is, the closer it is to the ideal dispersion curve in a perfect bonding state. Conversely, as the stiffness coefficient decreases, the thin film is assumed to be completely separated from the substrate, hence it converges to the dispersion curve of the lamb wave, which is a plate wave only within the thin film.

## 3. Experimental Setup and Method

### 3.1. Preparation of Specimen

Thin titanium film was deposited on a silicon wafer (100) using a DC magnetron sputtering equipment to derive the stiffness coefficient of the thin film through the dispersion simulation. The thickness of the thin film was deposited with a target of 600 nm. In the sputtering process, higher DC power increases the residual stress in the material and decreases the external load needed to fracture the interface, as well as the bonding force [20]. Therefore, in order to fabricate the specimens of different bonding strengths, the process was performed for five groups with different DC power levels, from 28.8 W to 144 W. Other deposition conditions were set as the same, except for DC Power. The other detailed deposition conditions are set at a chamber pressure of 5 Torr, a target distance of 7 cm, a flow rate of 15 sccm, and an operating pressure of 3.7 mTorr.

**Figure 3:**
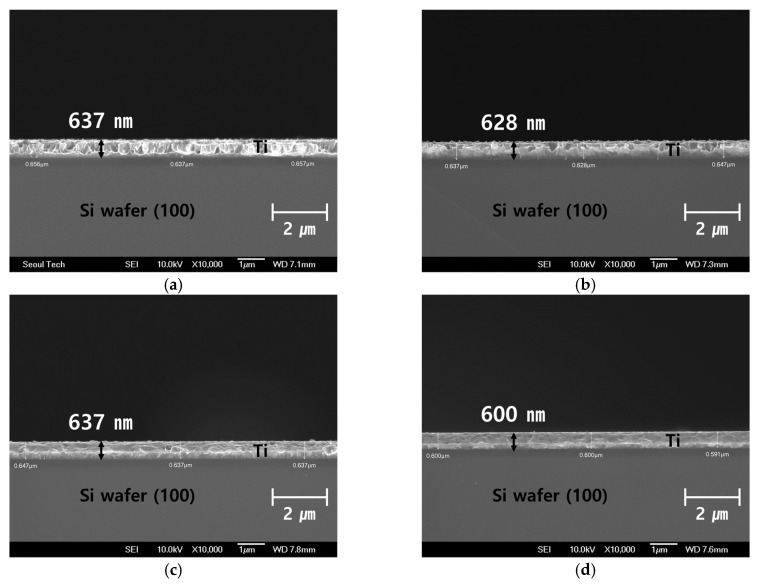

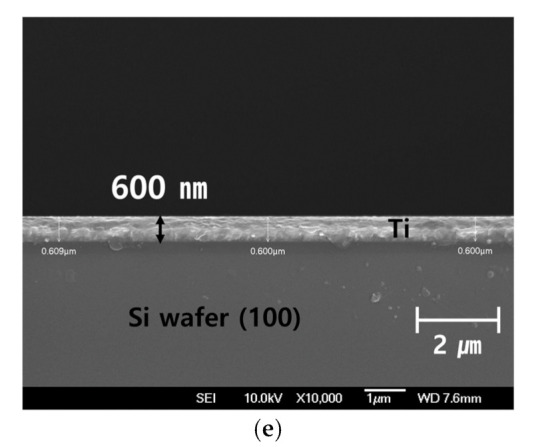
SEM image for Ti thin film thickness measurement: (**a**) 28.8 W; (**b**) 57.6 W; (**c**) 86.4 W; (**d**) 115.2 W; (**e**) 144 W.

Since the dispersion characteristics of the *SAW* propagating through the thin film is greatly affected by the thickness of the thin film, an accurate thickness was measured by taking a cross section of the thin film using a scanning electron microscope (SEM). The measurements are as shown in Figure 3. From the cross-section images, it was found that the error was about 4.5% on average when compared to the initial target thickness of 600 nm. It appears that the error in thickness occurred due to a large variation in deposition rate depending on the deposition conditions.

### 3.2. Surface Acoustic Wave Velocity Measurement Using a Scanning Acoustic Microscope

The velocity of the *SAW* was measured using the *Vz* method of scanning acoustic microscope, which is widely used for non-destructive evaluation of material properties. The *Vz* method calculates the velocity of the *SAW* by superimposing the signals incident/reflected vertically and the leakage signals of *SAW* that are incident on the specimen at a second critical angle along the lens shape and propagated to the surface. The waveform of the signal along the defocused distance of the probe lens is called a *Vz* curve, and the distance between signal peaks is defined as Δ*z*. The velocity of the *SAW* is defined as in Equation (9) below.

(9) $$C_{SAW}=C_{W}{\left[1-{\left(1-\frac{C_{W}}{2f△z}\right)}^{2}\right]}^{-1/2}$$

**Figure 4:**
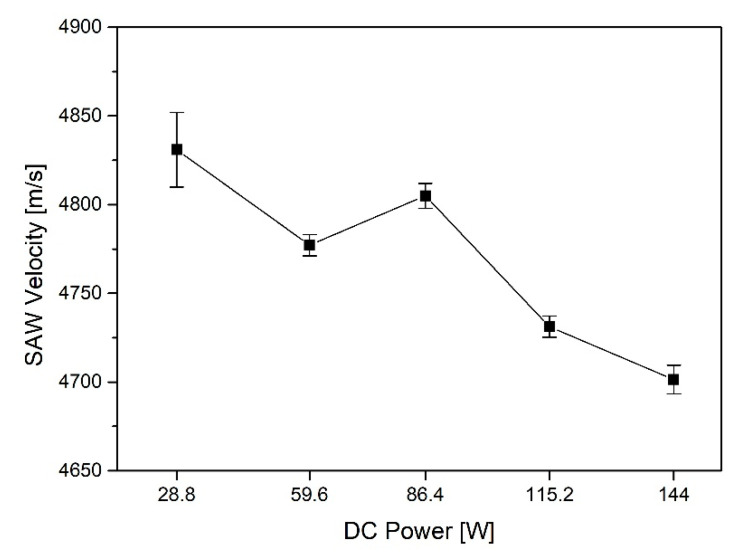
The results of *SAW* velocity using a scanning acoustic microscope (m/s).

Here, $C_{W}$ is the sound speed in the water, and *f* is the applied frequency. The *SAW* velocity was measured using a UH3 scanning acoustic microscope manufactured by Olympus, and a 200 MHz transducer was used as the applied frequency. The wave velocity was measured five times for each specimen, and the results are as shown in Figure 4. The averages of *SAW* velocity according to the test specimen production conditions are 4831 m/s at 28.8 W, 4777.2 m/s at 59.6 W, 4805 m/s at 86.4 W, 4731.2 m/s at 115.2 W, and 4701.4 m/s at 144 W.

As a result of measuring the *SAW* velocity of the Ti/Si specimen using a scanning acoustic microscope, it appeared that the higher DC power, the lower the wave velocity.

### 3.3. Evaluation of Stiffness Coefficient Using Dispersion Characteristics

**Figure 5:**
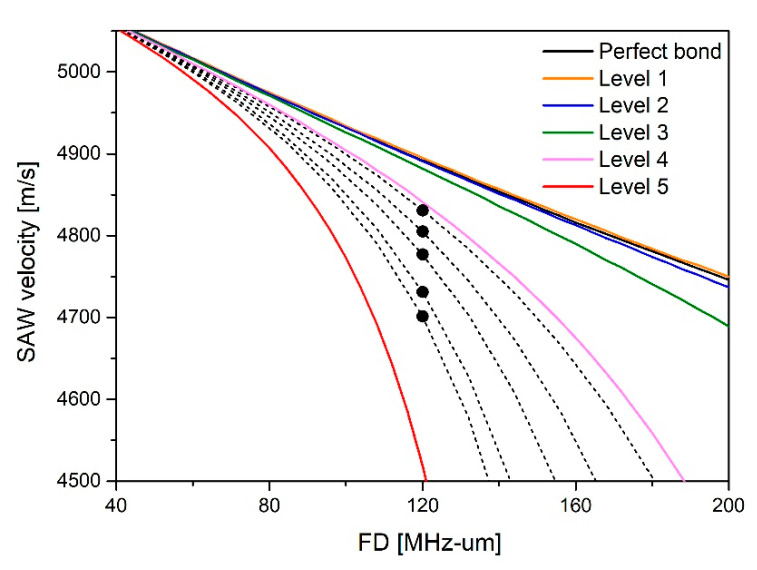
Results of *SAW* velocity on dispersion curve by interface condition.

The value of the stiffness coefficient for each specimen was quantitatively derived by comparing the dispersion curve according to the stiffness coefficient and the *SAW* velocity measured by the scanning acoustic microscope. On the dispersion curve divided by the 5 levels of stiffness coefficients, it was confirmed that the actual velocity of the *SAW* was distributed between the bonding levels 4 and 5. As a result of the simulation by subdividing the section, the stiffness coefficient was derived according to each velocity measurement value. The dispersion curves for each level are shown in Figure 5. In the figure, the dashed line means the subdivided dispersion curve and the stiffness coefficient corresponding to the measured *SAW* velocity can be derived. The results of the stiffness coefficients that correspond to the measured values of the *SAW* velocity of each specimen are as shown in Table 2.

### 3.4. Adhesion Force Evaluation by Nano-Scratch Test

NanoTest NTX, a commercialized nano-scratch equipment, was used to evaluate the quantitative adhesion force of the fabricated specimens. In the nano-scratch test, the critical load is the point where the thin film is completely debonded from the substrate and the work of adhesion is calculated by using the critical load [21]. The relationship between the work of adhesion and the critical load is as shown in Equation (10) below.

(10) $$L_{C}=\frac{\pi d^{2}}{8}{\left(\frac{2EW_{AD}}{t}\right)}^{1/2}$$

In the equation, $L_{C}$ is the critical load, *d* is the scratch width, $W_{AD}$ is the work of adhesion, and *E* and *t* are the elastic modulus and thickness of the thin film.

**Figure 6:**
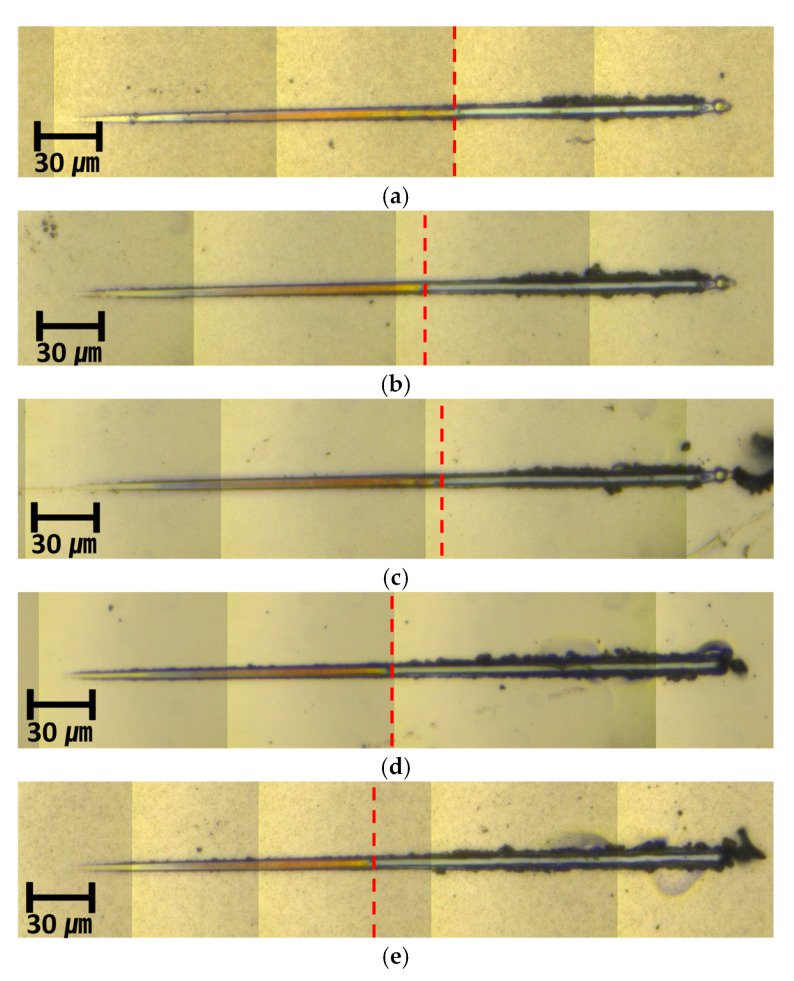
Nano-scratch test OM image: (**a**) 28.8 W; (**b**) 57.6 W; (**c**) 86.4 W; (**d**) 115.2 W; (**e**) 144 W.

In this study, since the same experimental conditions and materials were used, the work of adhesion is proportional to the critical load. Thus, the critical load measured from the nano-scratch test was expressed in terms of the adhesion force. The experimental conditions were set as shown in Table 3 below. The nano-scratch test was conducted five times for each specimen, and the load value at the point of interfacial failure was recorded by red dashed line with the optical microscope, as shown in Figure 6.

**Figure 7:**
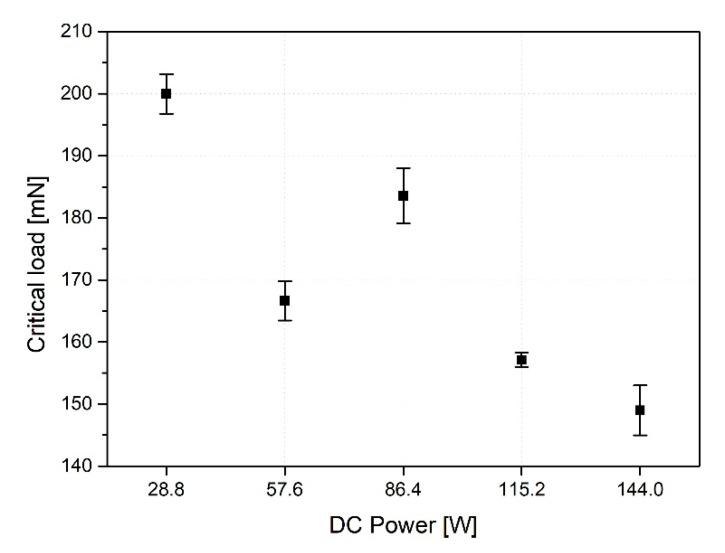
The results of critical load by nano-scratch test [mN].

As the results in Figure 7 indicate, the critical load tends to decrease when the DC power, a deposition condition, increases.

## 4. Conclusions

In this study, in order to evaluate the adhesion properties of thin film structures, the stiffness coefficients were derived by the dispersion characteristics of the *SAW*, and the reliability of the results was verified through the nano-scratch test. Table 4 shows the results of the stiffness coefficients from the dispersion characteristics and the results of the adhesion forces derived through the nano-scratch test. From the comparative evaluation of the stiffness coefficient and the adhesion force of the specimens according to the deposition condition, it was confirmed that the trends of the results were very similar. Through this, we confirmed the reliability of the dispersion characteristic simulation technique to evaluate the adhesion properties of thin films in this study.

## Figures and Tables

**Table 1:** Stiffness coefficient according to the bonding interface condition of multilayer thin film.

Interfacial Condition	Boundary Stiffness $10^{16\;}N/m^{3}$	Longitudinal Velocity (m/s)	Shear Velocity (m/s)
Perfect bond	Boundary continuity	-	-
Complete debonding	$K_{N}$$=0,\;K_{T}$ = 0	0	0
level 1	$K_{N}$$=216.0,\;K_{T}$ = 61.44	14,120	7530
level 2	$K_{N}$ = $54.0,\;K_{T}$ = 15.36	7060	3770
level 3	$K_{N}$ = $13.5,\;K_{T}$ = 3.84	3530	1880
level 4	$K_{N}$$=3.38,\;K_{T}$ = 0.69	1760	940
level 5	$K_{N}$$=0.84,\;K_{T}$ = 0.24	880	470

**Table 2:** The results of the stiffness coefficients according to deposition condition.

Specimen	DC Power Condition(W)				
	28.8	57.6	86.4	115.2	144
$K_{N}$$\;(10^{16}\;N/m^{3}$)	1.6065	1.1508	1.3125	1.0038	0.9429
$K_{T}$$\;(10^{16}\;N/m^{3}$)	0.459	0.329	0.375	0.287	0.269

**Table 3:** Experimental condition of nano-scratch test.

Tip Type	5 μm Sphero-Conical
Initial load (mN)	0.01
Final load (mN)	300
Loading rate (mN/s)	2.5
Speed (μm/s)	2
Length (μm)	350

**Table 4:** The compared result to the stiffness constants and the critical load.

Specimen		DC Power Condition (W)				
		28.8	57.6	86.4	115.2	144
$\mathrm{Stiffness}\;\mathrm{constant}\;(10^{16}\;N/m^{3})$	$K_{N}$	1.6065	1.1508	1.3125	1.0038	0.9429
	$K_{T}$	0.459	0.329	0.375	0.287	0.269
Critical load (mN)		199.95	166.61	183.57	157.11	148.99

## Data Availability

Not applicable.
